# Sex differences in the first impressions made by girls and boys with autism

**DOI:** 10.1186/s13229-020-00336-3

**Published:** 2020-06-16

**Authors:** Meredith L. Cola, Samantha Plate, Lisa Yankowitz, Victoria Petrulla, Leila Bateman, Casey J. Zampella, Ashley de Marchena, Juhi Pandey, Robert T. Schultz, Julia Parish-Morris

**Affiliations:** 1 Children’s Hospital of Philadelphia, Center for Autism Research, 2716 South St, Philadelphia, PA 19104 USA; 2 Department of Psychology, University of Pennsylvania, 3720 Walnut St, Philadelphia, PA 19104 USA; 3 Department of Psychology, University of the Sciences, 600 S 43rd St, Philadelphia, PA 19104 USA; 4 Department of Psychiatry, Perelman School of Medicine of the University of Pennsylvania, 3400 Civic Center Blvd, Philadelphia, PA 19104 USA; 5 Department of Pediatrics, Perelman School of Medicine of the University of Pennsylvania, 3400 Civic Center Blvd, Philadelphia, PA 19104 USA

**Keywords:** Autism spectrum disorder, First impressions, Sex differences, Camouflage

## Abstract

**Background:**

Individuals with autism spectrum disorder (ASD) are characterized by social communication challenges and repetitive behaviors that may be quickly detected by experts (Autism Res 10:653–62, 2017; American Psychiatric Association, Diagnostic and statistical manual of mental disorders, 2013). Recent research suggests that even naïve non-experts judge a variety of human dimensions using narrow windows of experience called “first impressions.” Growing recognition of sex differences in a variety of observable behaviors in ASD, combined with research showing that some autistic girls and women may “camouflage” outward symptoms, suggests it may be more difficult for naïve conversation partners to detect ASD symptoms in girls. Here, we explore the first impressions made by boys and girls with ASD and typically developing (TD) peers.

**Methods:**

Ninety-three school-aged children with ASD or TD were matched on IQ; autistic girls and boys were additionally matched on autism symptom severity using the ADOS-2. Participants completed a 5-minute “get-to-know-you” conversation with a new young adult acquaintance. Immediately after the conversation, confederates rated participants on a variety of dimensions. Our primary analysis compared conversation ratings between groups (ASD boys, ASD girls, TD boys, TD girls).

**Results:**

Autistic girls were rated more positively than autistic boys by novel conversation partners (better *perceived* social communication ability), despite comparable autism symptom severity as rated by expert clinicians (equivalent *true* social communication ability). Boys with ASD were rated more negatively than typical boys and typical girls by novel conversation partners as well as expert clinicians. There was no significant difference in the first impressions made by autistic girls compared to typical girls during conversations with a novel conversation partner, but autistic girls were rated lower than typical girls by expert clinicians.

**Limitations:**

This study cannot speak to the ways in which first impressions may differ for younger children, adults, or individuals who are not verbally fluent; in addition, there were more autistic boys than girls in our sample, making it difficult to detect small effects.

**Conclusions:**

First impressions made during naturalistic conversations with non-expert conversation partners could—in combination with clinical ratings and parent report—shed light on the nature and effects of behavioral differences between girls and boys on the autism spectrum.

In this paper, our terminology is drawn from World Health Organization definitions, such that the word “sex” refers to genetic makeup, and “gender” refers to a socio-cultural construct [1]; here, we use the words “girl” and “boy” to refer to biological sex. In line with preferences expressed by self-advocates and parents within the autism community [2–4], this paper uses both identity-first language (i.e., autistic girls and boys) and person-first language (i.e., girls and boys with autism) to reflect variability in language preferences.

## Background

Autism spectrum disorder (ASD) is a neurodevelopmental condition characterized by social communication deficits that are present during early childhood and persist into adulthood [5, 6].

Social impairments in ASD are linked to poor functional outcomes, including fewer friendships and higher rates of loneliness [7, 8], difficulties with romantic relationships [9, 10], reduced employment [11], and overall decreased quality of life [12, 13]. A significant number of referrals for autism evaluations are generated in schools, primary care settings, or therapeutic contexts where professionals must determine the need for expert assessment [14]. These interactions are often brief, and it is therefore important to understand how ASD presents during short windows of observation.

Recent research suggests that some individuals with ASD—and perhaps girls and women in particular—engage in effortful masking or camouflaging to hide their autism symptoms [15–24] and some develop compensatory behaviors designed to support social interaction [25]. While these compensatory behaviors may be effective in the short-term, maintaining them over time can be exhausting and distressing [24, 26]. There is a growing body of research on self-reported camouflage and compensation in adults, but less is known about how camouflage and compensation manifest in children. Understanding camouflage during the school years is critical because it may reduce the likelihood of being referred for an evaluation, thus contributing to the problem of delayed diagnosis—and delayed service onset—for autistic girls in particular [27]. Qualitative research has shown that long-term camouflaging is linked to poor mental health outcomes for individuals with ASD [17, 28] and identifying camouflage in childhood could provide opportunities to ameliorate these negative effects. Finally, it is essential to quantify the heterogeneity of autism—and how it may manifest differently in boys versus girls—in order to advance our understanding of this increasingly common condition and develop personalized treatments. In this study, we explore the first impressions made by boys and girls with ASD and typically developing (TD) peers during brief naturalistic interactions with new acquaintances.

### First impressions

“You never get a second chance to make a first impression.” This phrase may sound like folk psychology, but significant empirical evidence shows that adults and children [27–29] form rapid first impressions about new acquaintances—or “thin-slices”—that shape both immediate and long-term behaviors and attitudes [30, 31] and are rarely inhibited or corrected by deliberate thought processes [32]. First impressions of strangers predict the pursuit and intensity of future friendships [33], and negative first impressions create stigma towards a novel social partner [34], prompting behaviors like rejection and avoidance [35–37]. For individuals with neurodevelopmental conditions like ASD, the negative first impressions they make on other people can prove a stubborn obstacle on the path toward achieving social goals like friendships and positive functional outcomes like stable employment [38, 39]. While the majority of prior research on first impressions has utilized adult samples, these findings are largely replicated in studies with children [29–31, 34, 40].

Certain features reliably predict first impression quality (e.g., physical appearance [40] including clothing [41], shoes [42], and grooming habits [41]), but others are more subjective and vary according to demographic characteristics such as biological sex or race [43]. For example, previous research has shown that appearing extroverted and open to new experiences is positively related to dating success for male users of an online dating site, but not for female users [44]. Thus, some traits that confer a favorable impression for one demographic subgroup may have a null or inverse effect in another. This process could reflect the combined workings of cultural and societal biases, whereby people associate appearance-based and superficial behavioral cues with certain personality traits and social outcomes [45].

### First impressions in autism

Given a variety of social communicative differences that may be quickly evident for some individuals with ASD [46], such as atypical vocal prosody [47], unusual use of co-speech gestures [48], atypical facial expressivity [49], and general “awkwardness,” [50] many individuals with ASD face unique challenges when it comes to making positive first impressions [49, 51–60]. For example, several studies show that viewing a child actor displaying stereotypically autistic behaviors (e.g., rocking and hand flapping) leads to less positive judgments about the child, including fewer intentions to socially interact and lower ratings on a friendliness scale [52, 54, 58, 61–63]. Other research shows that negative first impressions of individuals with ASD can lead to social avoidance [53], reduced intentions to interact, and other forms of distancing [51, 55]. Research on first impressions made by children with ASD replicates patterns of negative evaluation seen in adults with ASD [47, 48, 50, 52–54, 57, 58, 60–63]. While there is evidence to suggest that diagnostic disclosure can improve the social evaluations of individuals with ASD, this research has been conducted exclusively with adult samples [64]. Additionally, prior studies of first impressions in ASD have limited generalizability due to small samples, typically 40 participants or less [50, 54, 59, 64]. One emergent area of research is focused on the “double empathy problem,” which posits that autistic individuals’ social interaction difficulties reflect a relational disjuncture in which experiential and neurological differences between TD and autistic people contribute to distinct social norms, expectations, and modes of understanding [65]. One implication of this framework is that autistic people may demonstrate enhanced social coordination with partners they perceive as more similar to themselves; thus, two individuals with ASD may relate better to one another than a dyad wherein one participant has autism and the other is typically developing.

**Fig. 1:**
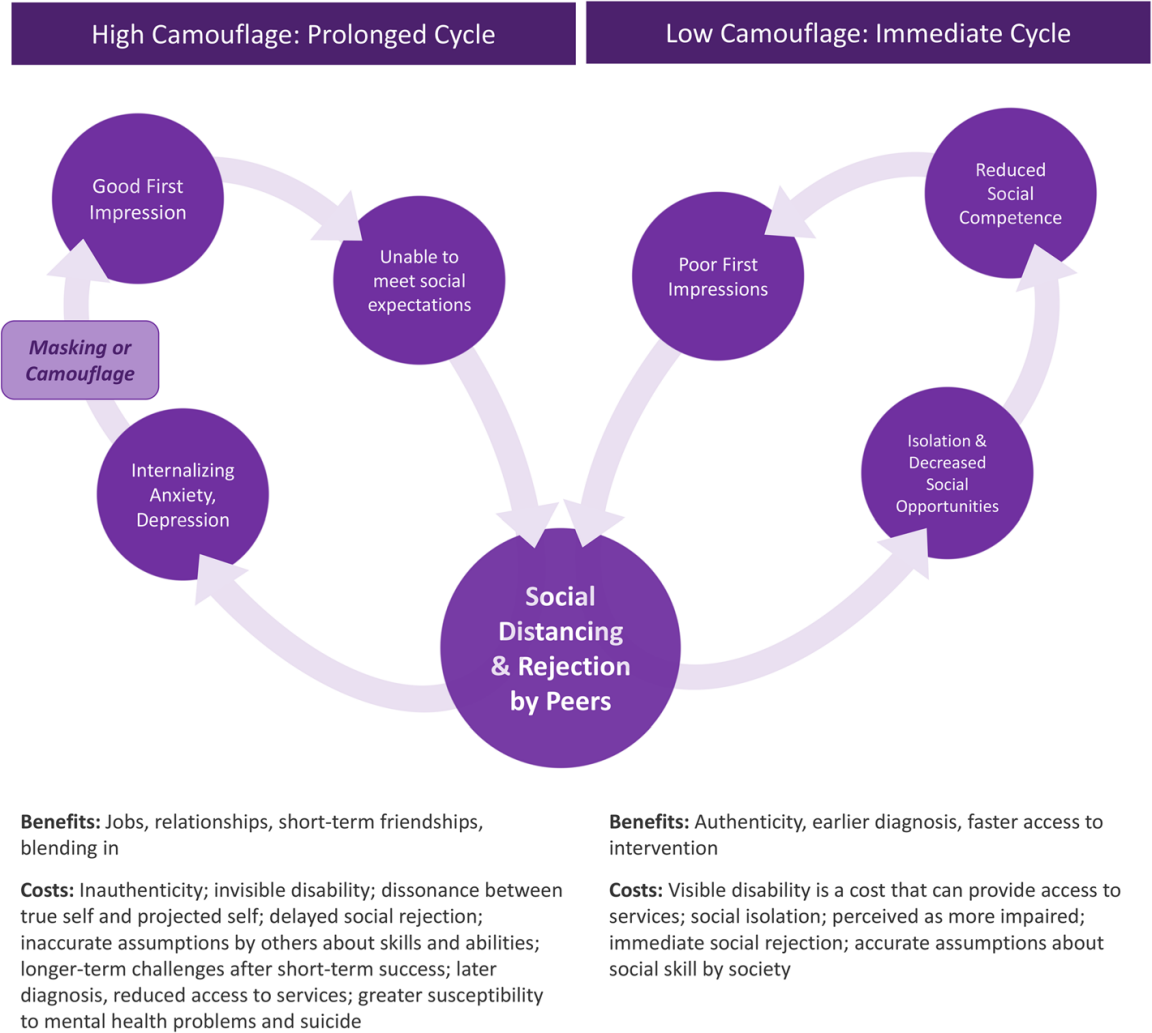
Cycle of negative first impressions in ASD, in high- and low-camouflaging contexts. The low camouflage cycle was informed by research demonstrating that individuals with ASD quickly make poor first impressions on peers [59]. The high camouflage cycle was informed by retrospective reports of camouflaging experiences by autistic women [23, 37].

Negative perceptions of individuals with ASD likely contribute to social exclusion in young adulthood [66], and reductions in the quantity and quality of social interactions have been argued to be most problematic for individuals who are consistently perceived pejoratively or inaccurately, such as those with ASD [50]. These cascading effects contribute to a negative cycle: TD peers perceive autistic individuals as having reduced social competence, and thus form poor first impressions [50]. These negative impressions lead to social distancing and rejection, which in turn create isolation and decreased social opportunities for the autistic individual. Diminished access to social opportunities may reinforce the perception that the autistic individual is less socially competent and reduces chances to practice social interaction, thus perpetuating the cycle (Fig. 1). For individuals who actively camouflage, a slightly different version of this cycle may occur over a longer period, with internalizing consequences: autistic individuals deploy camouflage to make good first impressions with TD peers, but as time goes on, they are unable to meet the associated social expectations. This leads to social distancing and rejection by peers, which in turn contributes to the development of internalizing issues (e.g., anxiety and depression). In order to reconnect socially, autistic individuals mask or camouflage their autism symptoms, and the cycle begins anew (Fig. 1).

### Research gap: sex differences

Researchers have demonstrated that autism is associated with poor first impressions [50, 58–60, 67], but a variety of autism-associated behaviors that impact first impressions have been shown to manifest differently in autistic girls versus boys. For example, from a distance, the social interactions of autistic girls may look similar to those of typically developing girls [15]. As adults, autistic women show greater discrepancies between the outward symptom expressions of ASD and their own internal experiences [37, 64], such that they report struggling with greater autism symptomology than is observed by others. Recent observational studies of language [16, 21], gesture [22], and social attention [66] further suggest that the behavioral symptoms of ASD may look different in girls and women than they do in boys and men. Consequently, first impressions of females with ASD may be significantly different—and perhaps more subtly atypical—compared to first impressions of males with ASD.

Prior studies on first impressions in ASD included few—if any—females and did not conduct separate analyses for the two sexes [49–51, 53, 56, 58–60, 63, 64, 68]. Thus, the current literature is unable to speak to potential sex differences in the first impressions made by individuals with ASD and skews the perceived first impression profile of ASD towards male-referenced patterns*.* However, evidence from the TD literature suggests that there are population-level sex differences in first impression formation (e.g., sex moderates the influence of otherwise identical features on first impressions [45, 51, 69]), raising the question of whether the first impressions of girls and boys with ASD may also differ from one another. Due to the paucity of research in this area, current social skills interventions that target improvements in first impressions are not sensitive to the potential moderating effect of sex, and it is unknown whether sex differences in first impressions could contribute to delayed diagnosis for autistic girls.

### The current study

The current study measures first impressions made by school-aged autistic girls and boys matched on IQ and social impairment as rated by both parents and clinicians, compared to TD boys and girls matched on IQ. Most prior research has studied non-expert first impressions using video-based ratings; here, we utilize a naturalistic conversation task to assess impression formation in a way that generalizes to everyday experiences, like meeting new people. Based on prior research suggesting that autistic individuals are perceived negatively compared to TD peers [47, 54–56, 59–62, 67] and that autistic girls may be more likely to engage in social camouflage or masking behaviors than autistic boys, and thus appear less affected to the naked eye [15, 18–23], we hypothesized that there would be an interaction between sex and diagnosis on first impression ratings. Specifically, we hypothesized that autistic girls would make a significantly better first impression than autistic boys on new conversation partners. In an exploratory analysis, we examined relationships between first impressions and clinical phenotype in boys and girls with ASD. Based on prior research suggesting that autistic girls may be more likely to engage in camouflaging behaviors than autistic boys [15, 18–23], we hypothesized that there would be a stronger correlation between ADOS scores and ratings of first impressions in autistic boys than in autistic girls. In this study, we conceptualized camouflage as the discrepancy in behavioral presentation as assessed by an expert clinician versus a naïve social partner.

## Methods

### Participants

Ninety-three participants with ASD and TD controls were selected from a larger study that included ASD diagnostic assessments, IQ testing, and behavioral tasks. ASD and TD groups were matched on IQ. Additionally, autistic girls and boys were matched on autism symptom severity as measured by the ADOS-2 [68] and SCQ [70]. Participant characteristics and matching statistics are provided in Table 1: see S1 for recruitment details and exclusion criteria. This study was overseen by the institutional review board at the Children’s Hospital of Philadelphia, and written informed consent was obtained from caregivers before study enrollment.

**Table 1:** Demographic and clinical characteristics of participants (means and standard deviations, in addition to minimum and maximum values)

	**ASD (*N*= 40)**		**TD (*N*= 53)**		**Effects**		
Sex ratio	15f, 25m (62.5% Male)		25f, 28m (53% male)		χ^2^ = .52, *p* = .47		
Race	Black/African American 5% (*n* = 2) White/Caucasian 85% (*n* = 34) Asian or Pacific Islander 2.5% (*n* = 1) Multiracial 5% (*n* = 2) Other 2.5% (*n* = 1)		Black/African American 22.6% (*n* = 12) White/Caucasian 60.4% (*n* = 32) Asian or Pacific Islander 3.7% (*n* = 2) Multiracial 13.2% (*n* = 7) Other 0% (*n* = 0)		χ^2^ = 9.68, *p* = .05		
Maternal education	High school or less 7.5% (*n* = 3) Bachelor’s or less 40% (*n* = 16) Graduate degree 50% (*n* = 20) Not reported 2.5% (*n* = 1)		High school or less 5.7% (*n* = 3) Bachelor’s or less 53% (*n* = 28) Graduate degree 42% (*n* = 22) Not reported 0% (*n* = 0)		χ^2^ = 1.27, *p* = .53		
	**Female**	**Male**	**Female**	**Male**	**Sex**	**Dx**	**Sex in ASD**
Age (years)	10.89 (2.30) 7.5–16.3	12.07 (3.27) 7.2–17.9	10.23 (2.70) 6.4–15.5	9.44 (1.89) 6.9–14.1	*p* = .99 *d* = .01	*p =* .002 *d* = − .69	*p* = .23 *d* = .40
Full-scale IQ	107.9 (11.23) 79–124	106.40 (13.68) 78–131	108.12 (12.46) 86–131	110.96 (12.43) 86–133	*p* = .70 *d* = .08	*p* = .30 *d* = .22	*p* = .72 *d* = − .12
Verbal IQ	105.80 (10.29) 85–122	106.20 (13.33) 83–130	109.04 (12.82) 80–128	109.21 (13.77) 86–131	*p* = .92 *d* = .02	*p* = .25 *d* = .24	*p* = .92 *d* = .03
Non-verbal IQ	107.80 (14.98) 80–130	105.32 (13.63) 78–130	104.76 (13.03) 81–129	108.54 (12.46) 82–130	*p* = .68 *d* = .09	*p* = .83 *d* = .05	*p* = .59 *d* = − .18
ADOS-2 CSS Total	6.60 (2.29) 3–10	7.00 (1.80) 2–10	1.08 (0.28) 1–2	1.32 (0.48) 1–2	*p* = .28 *d* = .10	*p* < .001 *d* = − 4.21	*p* = .54 *d* = .20
ADOS-2 SA CSS	6.53 (2.20) 3–10	7.36 (1.66) 3–10	1.44 (0.77) 1–3	1.75 (0.84) 1–3	*p* = .07 *d* = .17	*p* < .001 *d* = − 3.89	*p* = .19 *d* = .44
ADOS-2 RRB CSS	7.27 (1.75) 5–10	6.44 (2.14) 1–10	1.32 (1.11) 1–5	1.86 (1.92) 1–7	*p* = .94 *d* = -.01	*p* < .001 *d* = − 2.87	*p* = .22 *d* = − .41
SCQ Total	17.79 (7.39) 6–31	17.56 (7.88) 4–33	2.00 (2.24) 0–8	2.50 (3.05) 0–14	*p* = .86 *d* = .02	*p* < .001 *d* = − 2.87	*p* = .93 *d* = − .03

SCQ scores were missing for 1 ASD female. ADOS-2 *CSS* calibrated severity score, *SA CSS* Social affect calibrated severity score, *RRB CSS* Repetitive behaviors/restricted interests calibrated severity score. Chi-squared tests with Yates’ continuity correction tested for diagnostic group differences in sex ratio and maternal educational attainment. *P* values and Cohen’s d values for main effects of sex and diagnosis are shown, as well as *p* and Cohen’s d values of sex differences in the ASD group only

### Measures

All participants were administered the ADOS-2 [71], a clinician-administered assessment of the presence and severity of autism symptoms. Calibrated severity scores (CSS) were generated for the domains of Social Affect and Restricted and Repetitive Behaviors, as well as an overall score [72]. ADOS-2 calibrated severity scores of 1–2 indicate minimal-to-no evidence of autism symptoms, scores of 3–4 indicate a low level of autism symptoms, 5–7 a moderate level, and 8–10 a high level. The Social Communication Questionnaire (SCQ) [67] “Lifetime” version [73] was filled out by parents to assess the presence of ASD symptoms. To assess cognition, clinicians administered one of four cognitive tests; scores were standardized by J. Pandey and reduced to a single cognitive estimate, along with verbal and nonverbal subscores (see S2 for further details).

### Study procedure

Participants engaged in a five-minute “get-to-know-you” conversation with a young adult (confederate) whom they had never met. Conversation partners were seated across from one another at a small table. Prior to each conversation, study staff provided a close variant of the following prompt to the participant and confederate to introduce the task: “Alright, you two just chat and get to know each other. I’m going to finish getting a few things set up.” Confederates were unaware of the participant’s diagnostic status and the hypotheses of the study and were instructed to act as naturally as possible and not to dominate the conversation. Confederates included 21 undergraduate students or BA-level research assistants assigned to each participant based on scheduling availability (male *n* = 3; female *n* = 18; see S3 for additional details). Immediately after the conversation, confederates completed an extended version of the Conversation Rating Scale (CRS-E [71]; Additional file 2). For our primary analysis, the sum of all CRS-E questions was calculated (possible total score range = 6–42; (items 4 and 5 reverse coded)). CRS-E items were based on items from the Interpersonal Communication Satisfaction Inventory [70] and the Relational Communication Scale [72], two interpersonal communication rating scales that have been extensively validated in the communication literature [74].

### Statistical approach

Generalized linear models (GLM) modeled CRS-E scores using age, full-scale IQ, sex, and diagnostic group as predictors. The interaction between sex and diagnostic group was removed if not significant, and conditional main effects are reported in the absence of an interaction. GLM models were specified using the “Poisson” family for count data. Due to repeated confederates, generalized linear mixed effects models that included confederate ID and/or confederate sex were tested: results did not change (see S4 for additional details). To conserve degrees of freedom, simpler models without confederate factors are reported here. Tukey-corrected comparisons of estimated marginal means (EMM) were used to determine the exact nature of interactions. To assess the relationship between non-expert first impressions and expert clinical judgment of boys and girls with ASD, CRS-E scores were used to predict ADOS-2 calibrated severity scores (CSS) in the ASD group alone (after controlling for age and IQ). Effect sizes for GLM are reported as standardized mean differences (SMD) and Cohen’s d [75] for simple mean differences (e.g., Table 1). Spearman’s rho assessed the directionality of correlations within separate subgroups (e.g., boys with ASD and girls with ASD).

## Results

### Overall first impressions

**Fig. 2:**
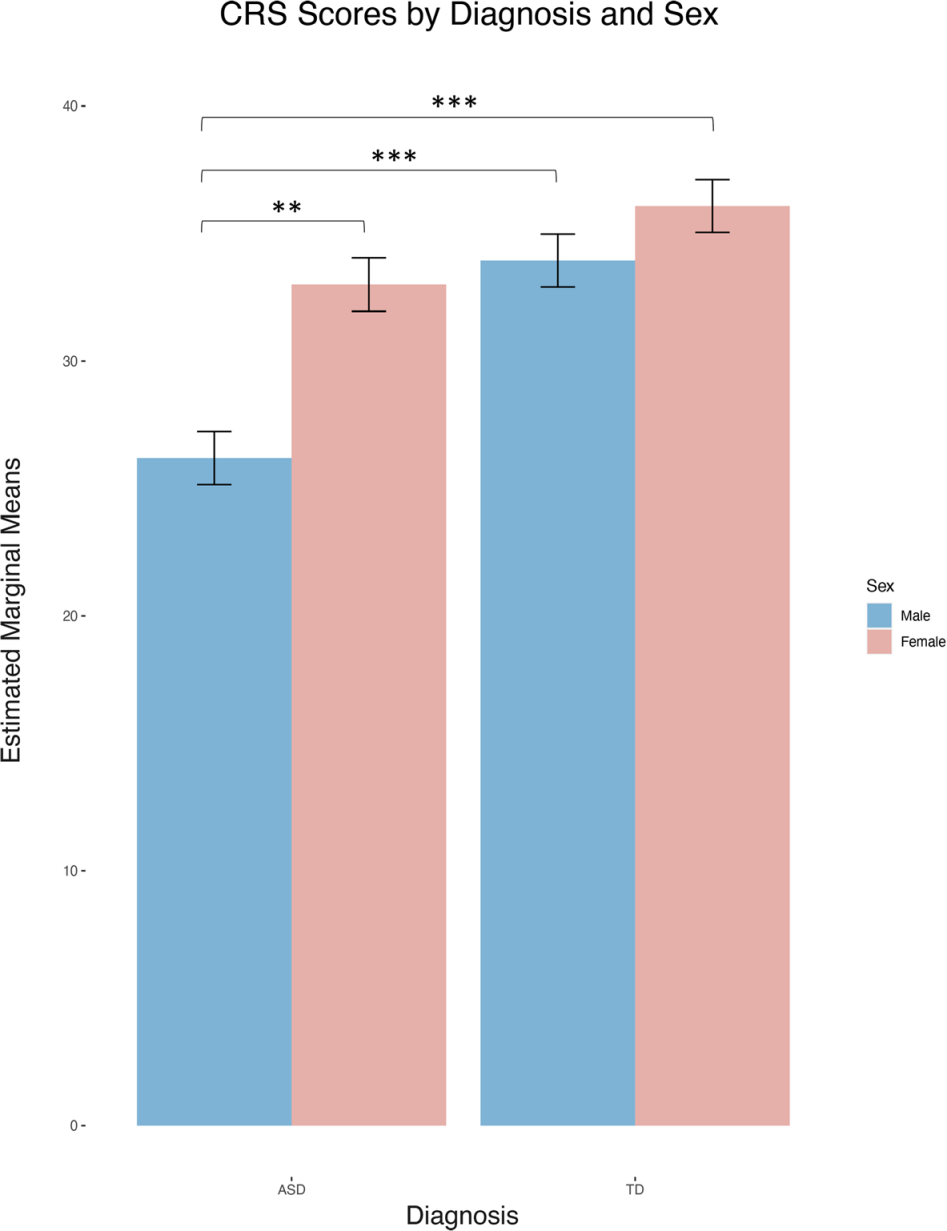
Estimated marginal mean scores on the Conversational Rating Scale–Extended (CRS-E) by diagnosis and sex (possible total score range = 6–42). Plot shows estimated marginal means of CRS-E total scores by diagnosis and sex. **p ≤ .001, ***p ≤ .0001

There was a significant interactive effect of sex and diagnosis on CRS-E total scores (estimate .19, *z* = 2.39, *p* = .02, SMD = 1.18; Fig. 2). Tukey-corrected pairwise tests revealed that boys with ASD received the lowest CRS-E total scores of any subgroup; boys with ASD were rated significantly lower than girls with ASD (estimate .23, *z* = 3.84, *p* = .0007, SMD = 1.26), lower than typical boys (estimate − .27, *z* = − 4.97, *p* < .0001, SMD = 1.29) and lower than typical girls (estimate − .32, *z* = -6.00, *p* < .0001, SMD = 1.38). Interestingly, there were no significant differences between the CRS-E total scores of autistic girls compared to either typical girls (estimate .08, *z* = 1.57, *p* = .40, SMD = 1.09) or typical boys (estimate .03, *z* = .45, *p* = .97, SMD = 1.03), who also did not differ from one another (estimate .06, *z* = 1.34, *p* = .54, SMD = 1.07; Fig. 2).

### Associations between first impressions and clinical phenotype

**Fig. 3:**
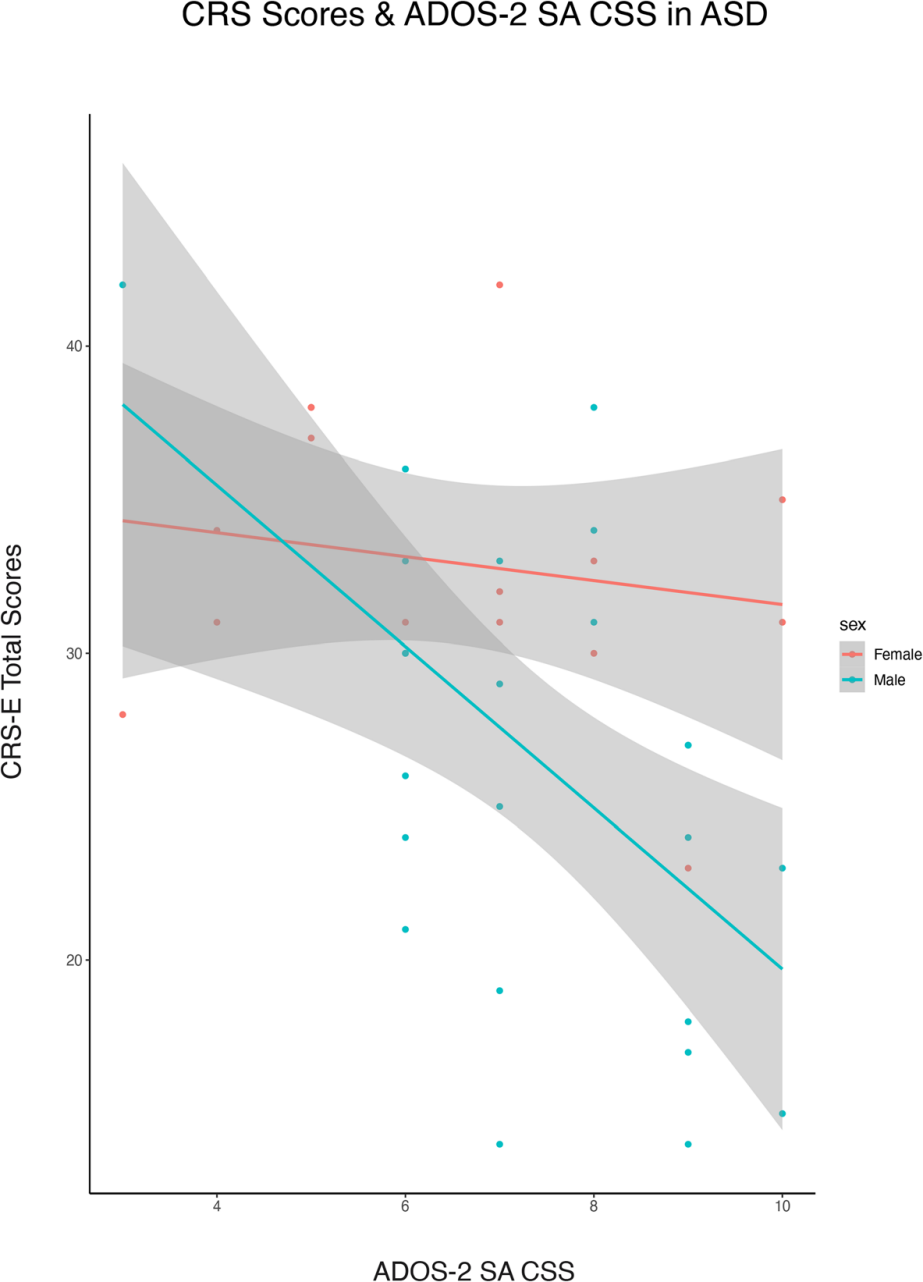
Correlation between CRS-E total scores and ADOS-2 Social Affect calibrated severity scores for autistic girls and autistic boys. Plot shows correlation between ADOS-2 Social Affect CSS and CRS-E Total scores. Logistic regression line is shown; bands represent 95% CI for the fit line

To assess the relationship between non-expert first impressions and expert clinical judgment of boys and girls with ASD, we used CRS-E scores to predict ADOS-2 calibrated severity scores (CSS) in the ASD group. After accounting for age and full-scale IQ across the entire ASD group, CRS-E total scores significantly predicted ADOS-2 Social Affect CSS scores (estimate = − .02, *z* = − 1.96, *p* = .05, SMD = .98), but did not predict ADOS-2 Total CSS (estimate = − .02, *z* = − 1.85, *p* = .06, SMD = .98) nor ADOS-2 Restricted Repetitive Behaviors CSS scores (estimate = − .001, *z* = − .17, *p* = .86, SMD = .99). This suggests that, overall, naïve raters form first impressions of social ability that are consistent with expert judgment, but the CRS-E does not capture variance associated with repetitive behaviors/restricted interests. Examining the relationship between CRS-E scores and ADOS-2 SA CSS scores in each sex separately, the relationship held for autistic boys (rho = − .50, *p* = .01), indicating that boys who made a poorer first impression on new acquaintances were also rated as more socially impaired by an expert clinician. Strikingly, this relationship was not present in girls (rho = − .14, *p* = .61), suggesting a disconnect between the first impressions made on new acquaintances and clinician ratings of social impairment in this subgroup (Fig. 3).

## Discussion

In this study, we found that school-aged autistic boys were judged similarly by naïve conversation partners and expert clinicians, whereas autistic girls were able to hide or mask their autism symptoms from new acquaintances and appear “typical” during brief “get-to-know-you” conversations. Previous research demonstrated that autistic individuals are perceived negatively compared to TD peers [50, 67, 76], but this is the first study to assemble a large enough sample of girls to examine sex differences [54, 58, 63, 64]. Our results suggest that prior research on the first impressions made by individuals with ASD may not generalize to autistic girls or women.

This study contributes to the literature by demonstrating—for the first time—that girls with ASD make significantly better first impressions on non-experts than boys with ASD. Thus, novel social partners may not be able to detect ASD symptoms or impairments in social functioning from brief interactions with girls [15, 17]. While this ability to “blend in” may benefit autistic girls in the short-term, it could also mean it is more difficult for teachers or pediatricians to identify when girls are struggling in social situations, and as such, they may be less likely to refer girls for diagnostic evaluations that could lead to evidence-based treatment and support [15]. In other words, these differences in autistic girls’ first impressions may lead to artificially low referral rates for girls, since they do not appear socially impaired “at first glance” and may contribute to chronic under diagnosis when adult observers do not immediately see cause for concern [19]. Autistic girls may therefore be less likely than autistic boys to receive critical supports shown to enhance long-term functioning [77]. Importantly, sex differences in patterns of social rejection by peers could also have meaningful implications for peer networks at school, which likely impact adult observers’ assessment of an individual’s social functioning [78, 79]. Finally, qualitative research suggests that long-term camouflaging is exhausting and is linked to poor mental health outcomes for individuals on the spectrum [17, 28], highlighting the costs of this potentially beneficial ability to “blend in” (Fig. 1).

The results reported here contribute to a growing literature that aims to sharpen our conception of ASD in girls by characterizing subtle differences in the first impressions made by girls versus boys. A practical clinical application of these results could include identifying new intervention targets that are personalized to each child’s profile of strengths and weaknesses (and are sensitive to the realities of gendered societal biases and expectations for girls), ultimately informing the way researchers evaluate treatment efficacy. For instance, it is possible that CRS-E scores capture relatively intact rapport building qualities in girls, which may be an area of strength that could be expanded upon in targeted social skills interventions.

It is important to highlight that girls and boys in our sample were equally affected by ASD symptoms (according to both clinician ratings and parent report), so the results of this study are unlikely to be driven by baseline sex differences in autism severity. Interestingly, we found that whereas social impairment and naïve first impressions were closely linked in autistic boys, they were not significantly related in autistic girls (i.e., correlations between CRS-E scores and ADOS-2 severity scores for ASD girls that were not significant). This lack of relationship in girls—despite a strong correlation in boys—is consistent with greater reports of effortful social compensation or masking by girls and women compared to boys and men on the spectrum [80]. Future research should explore whether girls are using specific strategies to maintain conversation—such as asking introductory questions about their conversation partner’s interests. If autistic girls pick up this tool or other strategies, it could normalize how they are perceived relative to typical peers, masking internal social struggles and serving as a form of “conversational camouflage.” Future research is warranted to explore the *intentionality* with which these camouflaging or compensatory behaviors are deployed—perhaps using self-report questionnaires, which have not yet been validated for children but have been used with adults. Interestingly, the results reported here could also be partially explained by differences in societal expectations of girls’ and boys’ social behavior, reflected in raters’ scoring patterns [81]. Understanding how a rater’s personal biases and sex-based expectations influence their perceptions of an individual with ASD is an important future research direction.

## Limitations

This study has significant strengths, including the largest sample of verbal girls with ASD in the first impressions literature and a well-matched TD control group, but it also has some limitations. Despite being one of the larger studies of autism in girls that utilizes direct behavioral assessment, the sample we report here is still unbalanced in that we have more autistic boys than girls, making it difficult to detect small effects; in addition, some of our subgroups were older or younger than others. We addressed this issue via statistical control, but it nonetheless warrants follow-up research with groups more closely matched on chronological age. Confederates (conversational partners) in this study were nearly all female, which limited our ability to assess patterns that might emerge during opposite-sex conversations in girls. Research shows that female raters are more forgiving when judging individuals with ASD, even when they are unaware of diagnostic status [82], suggesting that girls in our sample may have benefitted from gentler scoring. However, this does not explain why autistic boys still scored lower than any other group. One possibility relates back to the “double empathy problem”, wherein female non-autistic adults are better able to empathize with autistic girls than autistic boys due to experiential similarity (related to being female), and thus scored them less harshly [65]. Future studies with well-characterized raters of both sexes will explore how rater sex, biases, knowledge, and expectations about ASD may affect the way autism is perceived in males and females [64, 67].

In contrast to prior research that used third party observers or written vignettes to measure first impression formation [49, 50, 58–60, 64, 67], our study used a live interactional rating system. While the in-vivo rating method of this study is a strength, our measure of first impressions was somewhat blunt. The CRS-E includes only six questions, which restricted the range of possible domains we could assess. However, the use of a 7-point Likert scale resulted in distributions that were statistically variable and thus are more sensitive than a simple yes/no format [50].

It is important to note while that this study frames the ADOS-2 interaction as a measure of “real” autistic traits (i.e., expert clinical scores are presumed to be less susceptible to camouflaging behaviors than ratings by naïve confederates), other factors could also contribute to the observed pattern of differences. For example, it is possible that expert clinicians are simply better able to uncover girls’ ASD symptomatology; however, the observed pattern could also be attributed to different interaction lengths. That is, autistic girls may appear typical during a brief 5-minute conversation, but struggle to effectively camouflage their autistic behaviors for longer periods of time (e.g., an hour long ADOS-2 evaluation). Further research is necessary to parse the effects of exposure length, clinical expertise, and conversational context on camouflage in girls with ASD.

Characteristics of our study sample limit the generalizability of our findings. Participants were verbally fluent children aged 6–18 years old, so this study cannot speak to the ways in which first impressions may differ for younger children, adults, or individuals who are not verbally fluent. Understanding the developmental pathways that lead to more successful first impressions is key for developing appropriate interventions and supporting individuals who struggle with that skill, or who develop “autistic burnout” due to camouflaging [28]. Future research with larger samples of children and adolescents should explore the precise age at which sex differences in the use of camouflage might become apparent. Our diagnostic groups were not matched on race, with the TD group containing a larger number of racial minority participants. Prior US-based research shows that racial minorities are more likely to be rated negatively when measuring first impressions [34]. Although our results showed that TD participants were essentially at ceiling for their CRS-E scores, it is critical that future studies with very large samples be conducted with racially matched groups to examine potential differential effects of race on first impression formation in ASD. Finally, all of our confederates were young adults; given that some school-aged children with ASD may find it easier to interact with adults than peers, future research should include same-aged peers to determine whether interlocutor age has an effect on first impressions [51, 54, 83]. We did not evaluate participants’ impressions of the confederate or of the conversation as a whole. Understanding the perspective of the individual with ASD is an important missing piece that we are now addressing in follow-up research.

### Implications and future directions

This study has significant implications for understanding the kinds of impressions that children and adolescents with ASD make on people they meet for the first time. For example, the peer relationships of girls with ASD may be challenged when first impressions seem very typical, but pronounced autism symptomology emerges later. This discrepancy between first impressions and long-term behavior has implications for transforming acquaintances into lasting friendships, as well as for occupational success (e.g., autistic women have reported successfully getting jobs through good interviewing skills, but failing to maintain those jobs in the longer term as it becomes more and more exhausting to meet other people’s social expectations) [23, 39, 68]. Second, our finding that autistic girls produce typical first impressions has significant implications for ASD referral rates. The constellation of behaviors that lead to typical first impressions in girls remains largely unquantified (for examples of quantification see [16, 21, 22]). However, identifying those behaviors could increase the likelihood that providers are able to “see through” compensation or camouflage to identify girls in need of evaluation. Improved understanding of how autism manifests in girls and women—and the unique challenges they face—will enable researchers and clinicians to devise better and more personalized interventions and supports.

Given the vast heterogeneity that characterizes ASD, and our nascent understanding of how the gender spectrum interacts with biology and society, many future studies are possible. For example, there is virtually no research on first impressions in non-binary, gender diverse, or transgender individuals with ASD. Given that individuals with ASD have a greater-than-average likelihood of identifying as gender diverse [84–89] and that non-conforming or non-binary populations face specific societal challenges, it is urgent to understand this subgroup. Similarly, a multitude of demographic factors associated with appearance and behavior (e.g., race, ethnicity, cultural background, and bodily characteristics) contribute to first impression formation [34] and have yet to be examined in the context of ASD. Additional family- and environmental-level factors that contribute to social behavior should also be explored (e.g., parental gendered influence, social peer circle, and presence of non-autistic siblings). Future studies should also investigate how age-based social expectations could impact first impression ratings using well-powered cross-sectional and longitudinal samples. Finally, exploring time-linked effects of first impressions (e.g., at what point during an interaction do ASD girls start to diverge from TD girls/boys?) will provide critical information about how *long* objectively measured markers of camouflage can be maintained. Concurrent research on the physical and emotional toll of maintaining masking or camouflaging behaviors is important for helping girls and women achieve optimal outcomes.

## Conclusion

This study is the first to elucidate sex differences in the first impressions made by a relatively large, well-matched sample of children and adolescents with and without ASD. Our results suggest that autistic girls are perceived as more typical than autistic boys by naïve raters, despite comparable social impairment as measured by both clinician ratings and parent report. Thus, prior research demonstrating poor first impressions of individuals with ASD may be accurate for boys only and may not generalize to girls. Specifically, our finding that autistic girls’ first impressions do not correlate with clinician-rated social impairment adds to growing evidence that even when autistic girls and boys have commensurate levels of social disability, and girls may be perceived as functioning “better” by adults who observe them only briefly in a single social context.

## Supplementary information

**Additional file 1:** S1. Recruitment and exclusion criteria. S2. Measures. S3. Details of study administration. S4. Statistical approach.

**Additional file 2.** Conversation Rating Scale – Extended.

## Data Availability

The datasets generated and/or analyzed during the current study are not publicly available due to privacy concerns for minors with disabilities.

## Abbreviations

ADOS-2: Autism Diagnostic Observation Schedule–2nd edition
ASD: Autism spectrum disorder
EMM: Estimated marginal mean
GLM: Generalized linear model
IQ: Intelligence quotient
M: Mean
SCQ: Social Communication Questionnaire
SD: Standard deviation
SMD: Standardized mean difference
TD: Typically developing
