# Supplementary material for: Sex differences in the first impressions made by girls and boys with autism
Source: Mol Autism. 2020 Jun 16;11:49. doi: 10.1186/s13229-020-00336-3 (PMC7298946; doi:10.1186/s13229-020-00336-3)
Supplement: Supplementary file 1 — Additional file 1: S1. Recruitment and exclusion criteria. S2. Measures. S3. Details of study administration. S4. Statistical approach. [file 13229_2020_336_MOESM1_ESM.docx]

**S1. Recruitment and exclusion criteria.** Participants were recruited using a variety of methods, including public advertising, word of mouth, and re-recruiting from previous studies. Participants were excluded if they had a known genetic syndrome, history of concussion or brain injury that impacted current functioning, history of medication use that caused permanent changes in motor behavior, a gestational age below 34 weeks, or a primary language other than English. Parents of participants provided written informed consent to participate in this study, overseen by the Institutional Review Board of the Children’s Hospital of Philadelphia. ASD diagnoses were made by expert PhD-level clinicians using the clinical best estimate approach, with support from a research-reliable administration of the Autism Diagnostic Observation Schedule – Second Edition (ADOS-2^1^).

**S2. Measures.** All participants were administered the ADOS-2^1^, a clinician-administered assessment of the presence and severity of autism symptoms. ADOS-2 scores comprise two domains, Social Affect and Restricted and Repetitive Behaviors, which combine to create the overall score^2^. The Social Communication Questionnaire (SCQ^3^) “Lifetime” version^4^ was filled out by parents, typically the participant’s mother, prior to the study visit. The SCQ is a questionnaire-based screener for the presence of symptoms of ASD. To assess cognition, clinicians administered either the Differential Ability Scales-2^nd^ Edition (DAS-II^5^), the Wechsler Abbreviated Scale of Intelligence-2^nd^ Edition (WASI-II^6^), the Abbreviated Stanford-Binet Intelligence Scales-5^th^ Edition (SB5^7^), or the Wechsler Intelligence Scale for Children-5^th^ Edition (WISC-V^8^) which were standardized by J. Pandey and reduced to a single cognitive estimate, with verbal and nonverbal subscores.

**S3. Details of study administration.** Participants engaged in a five-minute “get-to-know-you” conversation with a novel adult (confederate) whom they had never met. Conversation partners were seated across from one another at a small table. Prior to each conversation, study staff provided a close variant of the following prompt to the participant and confederate to introduce the task: “Alright, you two just chat and get to know each other. I’m going to finish getting a few things set up.” Confederates were unaware of the participant’s diagnostic status and the hypotheses of the study, and were instructed to act as naturally as possible and not to dominate the conversation. Confederates included 21 young adult undergraduate students from Philadelphia area schools or BA-level research assistants (male *n* = 3; female *n* = 18). Confederates were assigned to each participant based on scheduling availability.

Immediately after the conversation, confederates completed an extended version of the Conversation Rating Scale (CRS^9^ see Appendix 1) as a measure of their first impressions. The CRS includes five questions indexing conversational interest, warmth, flow, boredom, and distance on a 1 to 7 Likert scale. Boredom and distance scores were reverse coded when calculating the total score, so that higher scores indicated better conversation, and better first impressions. The extended version of the CRS (CRS-E) includes an additional question about making appropriate eye contact, which was added to assess potential group differences specific to ASD. The sum of all CRS-E questions was calculated for our primary analysis (possible total score range = 6-42), and each CRS-E question was also examined separately to determine which features drove subgroup differences on CRS-E total scores.

**S4. Statistical approach.** Generalized linear models that included age, full-scale IQ, sex, and diagnostic group were used to predict CRS-E scores. The interaction between sex and diagnostic group was removed if not significant, and conditional main effects are reported in the absence of an interaction. Due to repeated confederates, generalized linear mixed effects models that included confederate ID and/or confederate sex were tested, but results did not change. To conserve degrees of freedom, simpler models without confederate factors are reported here. Models were constructed using the ‘stats’ package in R^10^, using family ‘Poisson’ due to the interval/count nature of CRS-E variables. Pairwise t-tests to determine the exact nature of interactions were conducted using the ‘emmeans’ package in R, with *p*-value adjustments using the Tukey method for comparing a family of four estimates. To assess relationships between CRS-E scores and clinical phenotype, we conducted generalized linear models (GLM) to predict CRS-E scores from scores on clinical measures (ADOS-2 CSS and domain scores), after controlling for age and IQ. Effect sizes for GLM are reported as standardized mean differences (SMD; appropriate for Poisson distributions and interpreted in standard deviations^11^), and Cohen’s d for simple mean differences (e.g., Table 1). Following Cohen^11^, SMD or d = 0.2 is considered a “small” effect, SMD or d = 0.5 a “medium” effect, and SMD or d = 0.8 a “large” effect. Spearman’s rho was used to assess the directionality of correlations within separate subgroups (e.g., boys with ASD, girls with ASD).

**References for Supplementary Materials**

1. Lord, C., Risi S, Bishop SL. Autism diagnostic observation schedule, second edition (ADOS-2). Torrance, CA: Western Psychological Services; 2012.

2. Hus V, Gotham K, Lord C. Standardizing ADOS Domain Scores: Separating Severity of Social Affect and Restricted and Repetitive Behaviors. *J Autism Dev Disord*. 2014;44(10):2400–24121.

3. Rutter M, Bailey A, Lord C. SCQ: The Social Communication Questionnaire. [Internet]. Los Angeles, CA: Western Psychological Services; 2003. Available from: https://www.wpspublish.com/store/Images/Downloads/Product/SCQ_Manual_Chapter_1.pdf

4. Chandler S, Charman T, Baird G, Simonoff E, Loucas T, Meldrum D, et al. Validation of the Social Communication Questionnaire in a Population Cohort of Children With Autism Spectrum Disorders. *J Am Acad Child Adolesc Psychiatry.* 2007 Oct;46(10):1324–32.

5. Elliott CD. Differential Ability Scales®-II - DAS-II [Internet]. San Antonio, TX: Harcourt Assessment; 2007. Available from: http://www.pearsonclinical.com/education/products/100000468/differential-ability-scales-ii-das-ii.html

6. Wechsler D. Wechsler Abbreviated Scale of Intelligence® - Second Edition (WASI®-II). San Antonio, TX: Pearson Clinical; 2011.

7. Roid GH. Stanford-Binet Intelligence Scales, Fifth Edition. Western Psychological Services; 2003.

8. Wechsler D. Wechsler Intelligence Scale for Children®-Fifth Edition (WISC®-V). San Antonio, TX: Pearson Clinical; 2014.

9. Ratto AB, Turner-Brown L, Rupp BM, Mesibov GB, Penn DL. Development of the Contextual Assessment of Social Skills (CASS): A Role Play Measure of Social Skill for Individuals with High-Functioning Autism. *J Autism Dev Disord*. 2011 Sep;41(9):1277–86.

10. R Core Team. R: A language and environment for statistical computing. Vienna, Austria: R Foundation for Statistical Computing; 2013. Available from: http://www.R-project.org/

11. Cohen J. Statistical power analysis for the behavioral sciences. 2. ed., reprint. New York, NY: Psychology Press; 2009. 567 p.
