# Supplementary material for: Sex differences in the first impressions made by girls and boys with autism
Source: Mol Autism. 2020 Jun 16;11:49. doi: 10.1186/s13229-020-00336-3 (PMC7298946; doi:10.1186/s13229-020-00336-3)
Supplement: Supplementary file 2 — Additional file 2. Conversation Rating Scale – Extended. [file 13229_2020_336_MOESM2_ESM.docx]

**Conversation Rating Scale – Extended**

The purpose of this questionnaire is to find out more about the conversation you just had. For each item, please circle a number, 1 through 7, to indicate to what extent you agree or disagree with the statement. Please complete all items.

| 1 = strongly disagree | 5 = agree somewhat |
| --- | --- |
| 2 = disagree | 6 = agree |
| 3 = disagree somewhat | 7 = strongly agree |
| 4 = neutral/unsure |  |

1. The other person was interested in what I had to say.

1 2 3 4 5 6 7

1. This person was warm and friendly.

1 2 3 4 5 6 7

1. The conversation flowed smoothly.

1 2 3 4 5 6 7

1. The other person acted bored by our conversation.

1 2 3 4 5 6 7

1. The other person created a sense of distance between us.

1 2 3 4 5 6 7

1. The other person made appropriate eye contact with me during our conversation.

1 2 3 4 5 6 7
